# Oxygen Transport in a Three-Dimensional Microvascular Network Incorporated with Early Tumour Growth and Preexisting Vessel Cooption: Numerical Simulation Study

**DOI:** 10.1155/2015/476964

**Published:** 2015-01-28

**Authors:** Yan Cai, Jie Zhang, Jie Wu, Zhi-yong Li

**Affiliations:** ^1^School of Biological Science and Medical Engineering, Southeast University, Nanjing 210096, China; ^2^Shanghai Pulmonary Hospital, Tongji University, Shanghai 200433, China; ^3^School of Naval Architecture, Ocean and Civil Engineering, Shanghai Jiaotong University, Shanghai 200240, China

## Abstract

We propose a dynamic mathematical model of tissue oxygen transport by a preexisting three-dimensional microvascular network which provides nutrients for an in situ cancer at the very early stage of primary microtumour growth. The expanding tumour consumes oxygen during its invasion to the surrounding tissues and cooption of host vessels. The preexisting vessel cooption, remodelling and collapse are modelled by the changes of haemodynamic conditions due to the growing tumour. A detailed computational model of oxygen transport in tumour tissue is developed by considering (a) the time-varying oxygen advection diffusion equation within the microvessel segments, (b) the oxygen flux across the vessel walls, and (c) the oxygen diffusion and consumption within the tumour and surrounding healthy tissue. The results show the oxygen concentration distribution at different time points of early tumour growth. In addition, the influence of preexisting vessel density on the oxygen transport has been discussed. The proposed model not only provides a quantitative approach for investigating the interactions between tumour growth and oxygen delivery, but also is extendable to model other molecules or chemotherapeutic drug transport in the future study.

## 1. Introduction

There are a lot of controversies about exactly how a tumour is initiated, but it is generally known that the interactions between tumour cells with the host microenvironment have played a predominant role in the pathophysiological mechanisms of tumourigenesis and progression. Hypoxia is believed to be one of the important hallmarks of the abnormal metabolic microenvironments in malignant tumours [1]. Since the diffusion limit of oxygen in tissue is 100–200 *μ*m [2], the regions far from blood vessels become hypoxic. However, many cancers growing in a blood-supply-sufficient environment, such as breast cancer and glioma, were also found to form the hypoxic region in foci. This suggests that tumour blood vessels fail to provide adequate levels of oxygen for the proliferating tumour cells. A key reason for this phenomenon is the abnormal architecture and function of tumour microvasculature, including the heterogeneous spatial distribution, the uneven vessel diameters, the leaky vessel wall, the poor blood perfusion, and the low level of red blood cell (RBC) velocity [3]. The heterogeneity of tumour blood flow due to the disorganized, dysfunctional microvasculature causes hypoxic region in tumour tissue despite the presence of blood flow.

Hypoxic tumour cells can express high levels of angiogenic regulators including vascular endothelial growth factor (VEGF). The new vasculature in response to the upregulated angiogenic factors provides essential nutrients for rapid neoplastic expansion of tumours [4]. Except this generally known mechanism of neovasculature, the “cooption” of preexisting vessel network also plays a significant role in early tumour progression [5]. Holash et al. [5] studied early angiogenic events using the rat C6 glioma model. The results showed that even the smallest C6 gliomas at just 1 week after implantation was well vascularized by coopting of preexisting blood vessels. Further experiments [6–8] revealed that when a small amount of tumour cells was implanted into healthy tissue, they managed to coopt and migrate along host vessels, as well as producing many chemical substances, such as VEGF, Ang-1, and Ang-2, to change the microenvironment around the host vessels, which can induce immature changes in the host tissue vasculature, including vessel dilation, increased capillary permeability, and tortuosity [9]. The local microenvironment especially the oxygen concentration distribution is important in the switch from preexisting vessel cooption to angiogenic vascularization. Detailed mathematical models are needed to provide an analysis framework not only to quantify physiological parameters such as tissue oxygen diffusion and vessel wall oxygen permeability that are not directly measurable, but also to handle the dynamic oxygen delivery processes.

Mathematical models for oxygen transport in capillary-perfused tissue have been studied extensively from last century. As a famous pioneering study, Krogh [2] presented a simple model to describe oxygen delivery through uniformly spaced parallel capillaries and considered a single capillary and its surrounding tissue as a cylinder region. Since it is difficult to measure the tissue oxygen concentration in three-dimensions with a micron resolution experimentally, more and more mathematical modellings and simulations have been proposed to obtain a better quantitative understanding in the microvascular oxygen transport. Goldman et al. published a series of work [10–12] on oxygen delivery in skeletal muscle, which have shown the importance of many features neglected in the Krogh model, including the capillary tortuosity and anastomoses, nonlinear oxygen consumption in tissue, myoglobin-facilitated diffusion and nonlinear oxyhemoglobin dissociation in the RBCs and plasma. Their models include a detailed description of intravascular resistance to oxygen transport and are capable of incorporating realistic three-dimensional microvascular network geometries. Secomb et al. [13] developed a theoretical model based on a Green's function approach, for realistic three-dimensional network geometries derived from observations of skeletal muscle, brain, and tumour tissues. However, the influences of haemodynamics conditions on the oxygen delivery are poorly considered in the above models.

For the modelling of oxygen delivery in tumours, oxygen concentration field is generally introduced to simulate the dynamic interactions of tumour cells with the surrounding tissues, by considering one or more pathophysiological characteristics during the process of malignant tumours growth. The reaction-diffusion equation is often used to describe the distribution of oxygen concentration. However, the source term of the equation is difficult to handle without the inclusion of preexisting vessels. Anderson [14] assumed that oxygen production is proportional to the density of extracellular matrix (ECM). Some other studies [15, 16] included the production of oxygen only from the simulation region boundary as constant oxygen source, while the oxygen can only be used by tumour cells and transported by diffusion from the boundary. In our previous coupled model [17], the oxygen supply by microvessels was assumed to be a linear function of local blood haematocrit, which is a rough way to deal with the oxygen transport through vessel network. These models may be useful for the study of tumour cell growth in a given nutrient environment; they cannot provide the dynamic oxygen supply variation caused by the preexisting vessel network adaptation to tumour growth and its influence on tumour growth.

The main aim of this study is to establish a dynamic mathematical model of tissue oxygen transport by a preexisting three-dimensional microvascular network which provides nutrients for an in situ cancer at the very early stage of primary microtumour growth. At the same time, the expanding tumour will consume oxygen during its invasion to the surrounding tissues and cooption of host vessels. In addition, the preexisting vessel cooption, remodelling, and collapse are modelled by the changes of haemodynamic conditions due to the growing tumour. Based on our previous haemodynamical calculation in solid tumour [18], we develop a detailed computational model of oxygen transport in tumour tissue by considering (a) the time-varying oxygen advection diffusion equation within the microvessel segments, (b) the oxygen flux across the vessel walls, and (c) the oxygen diffusion and consumption within the tumour and surrounding healthy tissue. The current model will provide a framework for analyzing the dynamic responses of local oxygen environment to the early avascular tumour growth and host vessel cooption, and will enable us to study quantitatively the influence of oxygen transport on the initiation of primary microtumours by preexisting vessel cooption.

## 2. Method

In this section, we first describe the generation of initial preexisting vessel network in the model (Section 2.1). The haemodynamic calculation through the 3D microvasculature is explained in Section 2.2. The oxygen transport as well as other chemicals is modelled based on the obtained haemodynamic information (Section 2.3). The tumour growth and microvessels are coupled by the changes of haemodynamic and chemical environments. It is based on the facts as follows: (a) the dynamic changes of tumour microvessels influence the chemical environments especially the oxygen supply, which determine the behavior of tumour cells (Section 2.4) and (b) the changes of haemodynamic and chemical environments due to the growing tumour induce tumour vessel dilation, cooption, remodelling, and collapse (Section 2.5). The simulation algorithm is summarized in Section 2.6.

### 2.1. Preexisting Vessel Network

**Figure 1:**
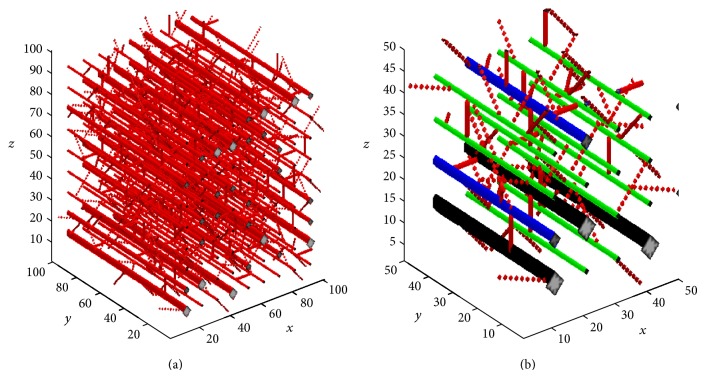
(a) 3D preexisting microvascular network with typical pattern of a normal arteriolar network, including parallel distributed vessels with varying vessel diameter and capillaries for cross link. (b) The enlarged view of local microvascular network. Different colours represent different orders of arterioles and capillaries (arterioles order 1: black; arterioles order 2: blue; arterioles order 3: green; capillaries: red).

For the morphological analysis we consider vessel segments within a cube simulation domain Ω of 1 mm^3^. A basic grid of 100 × 100 × 100 is generated uniformly in the cube with a distance of 10 *μ*m between the neighbouring nodes (Figure 1). The preexisting vasculature we designed in the basic model has a typical pattern of a normal arteriolar network. Parallel arterioles are distributed along *y* axis with uniform distance of 10 grids, that is, 100 *μ*m. Three orders with different vessel diameters are defined to reflect the heterogeneous characteristic of these arterioles. The vessel diameter and proportion of each order are shown in Table 1. In addition, capillaries are designed stochastically as cross links between the arterioles, having diameters of 8 *μ*m.

### 2.2. Haemodynamic Calculation

The haemodynamic model in this study is based on our previous work on the coupled modelling of intravascular blood flow with interstitial fluid flow [18, 22]. Briefly, the basic equation for the intravascular blood flow is the flux concentration and incompressible flow at each node. Flow resistance is assumed to follow Poiseuille's law in each vessel segment. The interstitial fluid flow is controlled by Darcy's law. The intravascular and interstitial flow is coupled by the transvascular flow, which is described by Starling's law. Blood viscosity is considered according to function of vessel diameter, local haematocrit, and plasma viscosity. In addition, vessel compliance and wall shear stress are correlated to vessel remodelling and vessel collapse, respectively, which will be explained in details in Section 2.5.

The main equations for blood flow calculation are as follows:

(1) $$\begin{array}{c} Q_{v}=\frac{\pi R^{4}\Delta P_{v}}{8\mu \Delta l},\\ Q_{t}=2\pi R\cdot \Delta l\cdot L_{p}\left(P_{v}-P_{i}-\sigma_{T}\left(\pi_{v}-\pi_{i}\right)\right),\\ Q=Q_{v}-Q_{t}, \end{array}$$

where *Q* is the flow rate of each vessel segment, which amounts to zero at each node of the vessel network due to the assumption of flux conservation and incompressible flow. *Q*_*v*_ is the vascular flow rate without fluid leakage; *Q*_*t*_ is the transvascular flow rate. Δ*l* and *R* are the length and radius of vessel segment. *P*_*v*_ and *P*_*i*_ are the intravascular pressure and the interstitial pressure of the vascular element. The total difference of *P*_*v*_ from plane *y* = 100 to *y* = 0 is set to be 3.5 mmHg as the driving force of blood in the network (or the boundary condition). *L*_*p*_ is the hydraulic permeability of the vessel wall. *σ*_*T*_ is the average osmotic reflection coefficient for plasma proteins; *π*_*v*_ and *π*_*i*_ are the colloid osmotic pressure of plasma and interstitial fluid.

The velocity of intravascular and interstitial flow satisfies

(2) $$\begin{array}{c} U_{v}=\frac{Q}{\pi R^{2}},\\ U_{i}=-K\nabla P_{i},\\ \nabla \cdot U_{i}=\frac{L_{p}S}{V}\left(P_{v}-P_{i}-\sigma_{T}\left(\pi_{v}-\pi_{i}\right)\right), \end{array}$$

where *U*_*v*_ and *U*_*i*_ are the intravascular flow velocity and the interstitial flow velocity, respectively; *K* is the hydraulic conductivity coefficient of the interstitium; *S*/*V* is the surface area per unit volume for transport in the interstitium.

The distribution of red blood cells (RBCs) at microvascular bifurcation is calculated based on the approach proposed by Pries and Secomb [23]. The details of blood rheology simulation were described in Wu et al. [22].

From the haemodynamic simulation, we are able to obtain the intravascular flow velocity *U*_*v*_ and the hematocritic *H* in the microvessel network which are used in oxygen concentration calculation and vessel diameter which is used to estimate vessel remodelling and collapse.

### 2.3. Oxygen Concentration Calculation

The glioma cell and endothelial cell behaviours are coupled by the changes of the chemicals in the extracellular matrix (ECM), such as oxygen and matrix degradation enzymes (MDEs). The transport of these chemicals (oxygen and MDEs) is modelled by quasisteady reaction-diffusion equations. The ECM is treated as a continuum substance and can be degraded by MDEs, while the MDEs are governed by diffusion, produced by TCs and ECs, and decay of itself. The equations describing the interactions of TCs and ECs with ECM and MDE are

(3) $$\begin{array}{c} \frac{\partial C_{f}}{\partial t}=-\delta C_{m}C_{f},\\ \frac{\partial C_{m}}{\partial t}=D_{m}\nabla^{2}C_{m}+\mu_{T}{\text{TC}}_{i,j}+\mu_{E}{\text{EC}}_{i,j}-\lambda C_{m}, \end{array}$$

where *C*_*f*_ and *C*_*m*_ are the ECM and MDE concentration, separately. The TC_*i*,*j*_ and EC_*i*,*j*_ terms represent a tumour cell and an endothelial cell located at a node position (*i*, *j*). Their values are either 1 if a cell is present or 0 if it is not. *D*_*m*_ is MDE diffusion coefficient, and, *δ*, *μ*_*T*_, *μ*_*E*_, and *λ* are positive constants.

To obtain more realistic oxygen concentration field, the advection and diffusion of oxygen in the vessel network are introduced [20]. The computational space is separated into three domains to characterize three distinct physiological processes, which are the oxygen advection equation inside the vessel, the oxygen flux across the vessel wall and the free oxygen diffusion in the tissue.

Specifically, the oxygen transport inside the vessel is represented by the advection equation

(4) $$\begin{array}{c} \frac{\partial C_{\text{o}\_\text{F}}}{\partial t}=-\overset{⃑}{U_{v}}\cdot \nabla C_{\text{o}\_\text{F}},\\ \frac{\partial C_{\text{o}\_\text{B}}}{\partial t}=-\overset{⃑}{U_{v}}\cdot \nabla C_{\text{o}\_\text{B}}, \end{array}$$

where *C*_o_F_ and *C*_o_B_ are the free and bounded oxygen concentrations, respectively. $\overset{⃑}{U_{v}}$ denotes the intravascular blood velocity.

The equation between the hemoglobin bound oxygen and free oxygen is

(5) $$\begin{array}{c} C_{\text{o}\_\text{B}}=4H\cdot C_{\text{Hb}}\cdot {\text{SO}}_{2}\left(C_{\text{o}\_\text{F}}\right), \end{array}$$

where *H* denotes haematocrit obtained from the haemodynamic calculation; *C*_Hb_ is the haemoglobin concentration within a red blood cell; SO_2_(*C*_o_F_) is the haemoglobin oxygen saturation.

The free oxygen flux across the vessel wall satisfies Fick's law:

(6) $$\begin{array}{c} \frac{\partial C_{\text{o}\_\text{F}}}{\partial t}V=J\cdot A, \end{array}$$

where *V* and *A* are the tissue volume and the associated vessel wall area. *J* is the oxygen flux which is obtained by

(7) $$\begin{array}{c} J=-L_{p}\frac{\left(C_{\text{o}\_\text{F}}^{\text{ex}}-C_{\text{o}\_\text{F}}^{\text{in}}\right)}{\alpha w}, \end{array}$$

where *C*_o_F_^ex^ and *C*_o_F_^in^ are the free oxygen concentrations at the exterior and interior surfaces of the vessel, respectively; *α* is the Bunsen solubility coefficient; *w* is the vessel wall thickness. *L*_*p*_ is the vessel wall permeability which is varied in different maturity level of vessel segments.

The interstitial fluid velocity is very slow due to the low interstitial pressure gradient in the tumour region. In fact, *U*_*i*_ is almost 100 times smaller than *U*_*v*_ in value according to the simulation results in our previous model. Therefore, we assume the free oxygen transported through the tissue space is governed by the oxygen diffusion equation which is not influenced by the interstitial fluid velocity *U*_*i*_.

Consider

(8) $$\begin{array}{c} \frac{\partial C_{\text{o}\_\text{F}}}{\partial t}=\nabla \cdot \left(D_{\text{o}}\nabla C_{\text{o}\_\text{F}}\right)-\gamma {\text{TC}}_{i,j}, \end{array}$$

where *D*_o_ is the tissue oxygen diffusion coefficient and *γ* is the consumption coefficient.

The initial condition of ECM density is set to be 1 and other chemicals' concentrations (oxygen and MDEs) are 0. No-flux boundary conditions are used in the simulation field. Since chemicals are transported much faster than the characteristic time for cell proliferation and migration, the chemicals' concentrations are solved to steady state at each time step of the simulation with an inner iteration step of 5 s.

### 2.4. Tumour Cell Phenotype

The probabilistic hybrid model for tumour cell growth is based on the previous work [17]. The 3D model is defined on a 100 × 100 × 100 grid to cover a 1 mm × 1 mm × 1 mm volume, so the grid length corresponds approximately to the size of a tumour cell, that is, 10 *μ*m.

We assumed three different phenotypes of glioma cells: the proliferating cells (*P*), the quiescent cells (*Q*), and the necrotic cells (*N*). Initially, we put 20 proliferating cells in the central area. Two thresholds of oxygen concentration for cell proliferation (*θ*_prol_) and cell survival (*θ*_surv_) are introduced to describe the effects of oxygen field on the tumour cell actions. To be specific, if there is enough oxygen (*C*_o_ ≥ *θ*_prol_) and a space available, a tumour cell will proliferate to two daughter cells with a probability, defined as *T*_age_/*T*_TC_. *T*_age_ is the tumour cell age range from 1 to *T*_TC_ and with an incremental 1 in each simulation time step. *T*_TC_ is the tumour cell proliferation time (set to be 9 hour, that is, 6 time steps). This assumption means an older tumour cell would be more likely to proliferate. One of the two daughter cells will replace the parent cell and the other cell will move to the neighbouring element. When the local oxygen concentration at a tumour site is below the cell survival threshold *θ*_surv_, the tumour cell is marked as necrotic cells and will not be revisited at the next time step. The necrotic cells have a probability of 20% to disappear to form available space for glioma cells and endothelial cells if they stay necrotic for more than 45 hours (30 time steps). When tumour cells satisfy the survival condition but there is no neighbouring space for them to proliferate, they will go to quiescent.

Each phenotype of tumour cell has different coefficients of the consumption rate of oxygen and the production rate of MDEs [17] (Table 2).

### 2.5. Vessel Cooption, Remodelling and Collapse

Experimental and clinical studies both revealed that microvessel diameter increases in response to growth factors. Döme et al. [9] found that even a single tumour cell can induce radical changes in the host tissue vasculature in a mouse model of glomeruloid angiogenesis. In our model, we consider vessel dilation as the first signal of a preexisting vessel becomes an immature vessel. A vessel segment surrounded by active tumour cells will increase its radius *R* with the rate 0.4 *μ*m/h if *R* ≤ *R*_max⁡_ = 20. At the same time, the permeability of vessel wall *L*_*p*_ is increasing in a dilation vessel and satisfies

(9) $$\begin{array}{c} L_{p}=L_{p}^{T}\left(1-\frac{R_{\max}-R}{R_{\max}}\right), \end{array}$$

where *L*_*p*_^*T*^ represents the vessel permeability value in tumour tissue.

For a preexist vessel, once vessel dilation occurs, the vessel segment was treated as an immature vessel and was allowed to vary in radius in response to the difference between intravascular pressure and interstitial pressure. The capillary compliance satisfies the empirical equation of Netti et al. [21]:

(10) $$\begin{array}{c} R=\left\{\begin{array}{cc} R_{0}{\left(\frac{P_{v}-P_{i}+P_{c}}{E}\right)}^{b}, & \text{immature}\;\text{vessel},\\ R_{0}, & \text{mature}\;\text{vessel}, \end{array}\right. \end{array}$$

where *R*_0_ is the origin radius of the capillary; *b* is the compliance exponent; *E* is the compliance coefficient. *P*_*c*_ is the collapse pressure and represents the ability of a vessel segment remaining structure intact under the pressure difference.

Based on the above equation, when the immaturation level of the vessel segment become more serious, the *P*_*i*_ increases due to the higher *L*_*p*_, which can induce vessel compressing. When vessel radius is compressed to below zero, the vessel segment collapses. Another factor leading to vessel collapse in tumours is the apoptosis of endothelial cells due to the disturbed blood flow or severe reduction of blood flow rate in the vessel. Wall shear stress (WSS) is used to estimate this kind of vessel regression, similar to our previous work [17]. The WSS of a vascular segment *k* can be calculated as

(11) $$\begin{array}{c} \tau =\frac{\Delta P_{V}\cdot R}{2\Delta l}. \end{array}$$

We assume that a circulated vessel, which is surrounded by the TCs, will collapse with a predefined probability if the WSS value in the vessel is less than a threshold *τ*_crit_ = 0.5*f*_0_ (*f*_0_ is the mean WSS value of the vessels of Arterioles order 3). The probability is assumed to be proportional to the duration of low WSS in the vessel; that is, the longer the vessel experiences the low WSS, the more likely the vessel is to collapse if the criterion is satisfied.

### 2.6. Simulation Algorithm

*Step 0. *Initialize. 

*Step 0.1*. Create the 3D preexisting vessel network and initialize the model parameters. 

*Step 0.2.* Put 20 proliferating cells with random ages from *T* = 1 to *T*_TC_ in the central area. 

*Step 0.3.* Set up fluid flow boundary conditions. 

*Step 1*. Haemodynamic calculation (see (1)–(2)), and obtain the flow information. 

*Step 2*. Calculate the chemical's concentration field. 

*Step 2.1*. Solve (3) to obtain the concentration distribution of ECM and MDE. 

*Step 2.2*. Solve (4)–(5) to obtain the oxygen transport inside the vessel using the intravascular blood velocity $\overset{⃑}{U_{v}}$ and haematocrit *H* from Step 1. 

*Step 2.3*. Solve (6) and (7) to obtain the free oxygen flux across the vessel wall. 

*Step 2.4*. Solve (8) to obtain the free oxygen transported through the tissue space. 

*Step 2.5*. Update the oxygen concentration field through the simulation domain. 

*Step 3*. Determine the behavior of tumour cells according to the local oxygen concentration, the available space, and the cell age and update the tumour cell distribution. 

*Step 4*. Update the vessel network. 

*Step 4.1*. Vessel cooption and vessel radius and permeability changes (see (9)) in immature vessels. 

*Step 4.2*. Vessel remodelling according to (10) update the radius of vessel segments. 

*Step 4.3*. Certain vessel collapse based on the *R* changing and WSS criterion (see (11)). 

*Step 5*. Go to Step 1.

Each time step increment (*T* = *T* + 1) corresponds to 1.5 hour. We will use nondimensional time unit instead of hours in the following results. All parameters used in the simulation are summarized in Table 3.

## 3. Results

### 3.1. Oxygen Concentration Distribution with Early Tumour Growth

**Figure 2:**
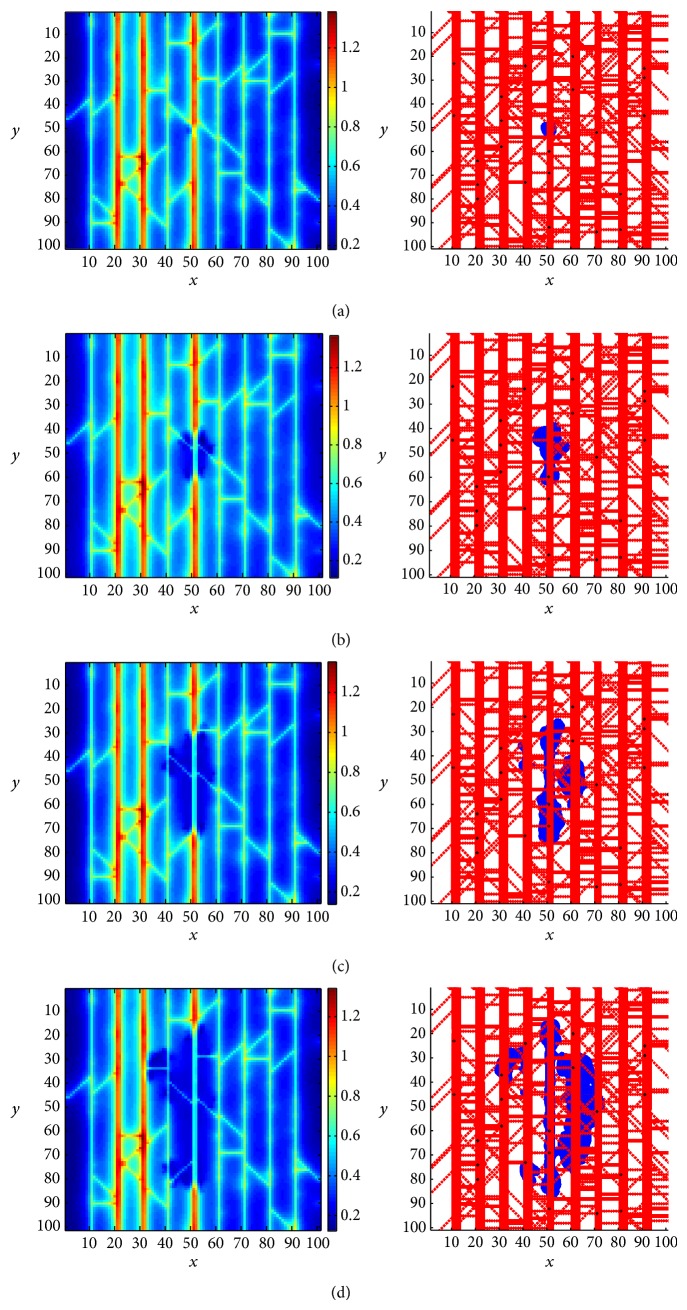
The distributions of oxygen concentration (left column) at plane *z* = 50 and the tumour invasion region (right column) from *x*-*y* view, at *T* = 10 (a), *T* = 50 (b), *T* = 100 (c), and *T* = 150 (d), respectively.

Figure 2 shows the dynamic processes of oxygen transport during the tumour growth and invasion. The blue regions shown in the right column represent the tumour invasive areas. It is found that the tumour cells generally grow along the parallel arterioles of the preexisting vessel network, which can provide sufficient oxygen for tumour growth. At the same time, the growing tumour undergoes central necrosis, due to the oxygen consumption of proliferating tumour cells, resulting in the hypoxia in the tumour invasion region (shown in the left column in Figure 2). In the current model, the vessel cooption and remodelling are also considered as key factors to control the dynamic interactions between preexisting vessels and growing tumour. With the cooption and remodelling of preexisting vessels induced by the invasion of tumour cells, some microvessels become immature and surrounded by tumour cells to be intratumour vessels. Due to the compression of tumour cells on the vessel wall and the continuous reduction of WSS, these immature vessels (especially capillaries between arterioles) will undergo collapse. As a consequence, the surrounded tumour cells become necrotic and will invade to healthy tissue further, increasing the tumour invasive area and forming some clusters of surviving tumour cells (shown in the right column in Figure 2).

### 3.2. Sensitivity to Preexisting Vessel Network

**Figure 3:**
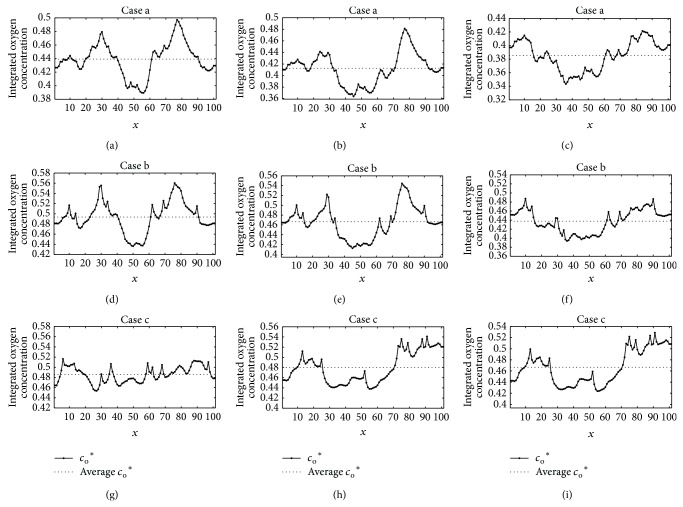
Distribution of integrated oxygen concentration *c*_o_^*^ along *x*-axis at plane *z* = 50 in three cases of different initial MVD. The dotted lines represent the average oxygen concentrations. Case b: the baseline model ((d) *T* = 50; (e) *T* = 100; (f) *T* = 150); Case a: a MVD value of 0.5 time of that in case b ((a) *T* = 50; (b) *T* = 100; (c) *T* = 150); Case c: a MVD value 1.5 times larger than that in case b ((g) *T* = 50; (h) *T* = 100; (i) *T* = 150).

To access the sensitivities to the preexisting vascular network, we changed the microvessel density (MVD) to values of 0.5 time (Case a) and 1.5 time (Case c) of that in the baseline case (Case b). The distribution of integrated oxygen concentration *c*_o_^*^ along *x*-axis at plane *z* = 50 in three cases is shown in Figure 3. The dotted lines show the average oxygen concentrations through the plane. In the three cases, the average oxygen concentrations increase with the initial MVD and decrease with tumour growing. In addition, the reduction of the average *c*_o_^*^ is more significant in the less MVD case (Case a) than that in the baseline (Case b) and more MVD (Case c) cases, which suggests that the emergence of hypoxia-induced tumour angiogenesis will be targeted earlier in a blood-supply-deficient microenvironment.

The oxygen concentration at the central region around *x* = 50 is always below the average *c*_o_^*^, which corresponds to the hypoxia in the tumour center. The hypoxia region will gradually expand with tumour growth, while the concentration gradient of oxygen will reduce at the same time. This indicates the oxygen concentration distribution in the modelling region towards equilibrium in the dynamics of oxygen consumption of tumour growth and oxygen production of preexisting vasculature. It is noteworthy that the oxygen distribution remains substantially unchanged after *T* = 100 in Case c (Figures 3(h) and 3(i)), which suggests the balance between oxygen consumption and production emerged earlier in a blood-supply-sufficient microenvironment.

### 3.3. Flow-Dependent and Flow-Independent Oxygen Transport

**Figure 4:**
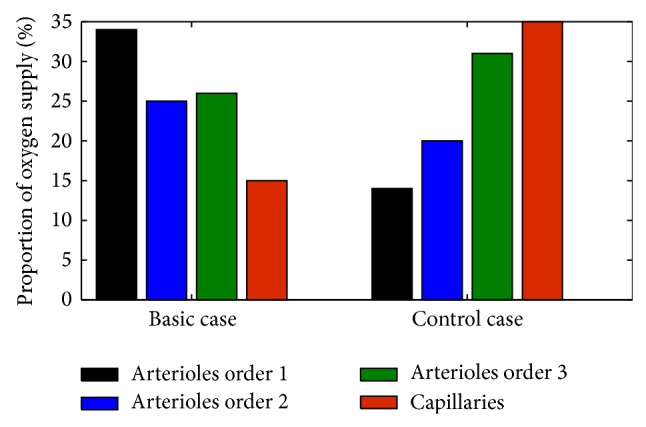
The proportion of oxygen supply to the tumour tissue by every arterioles order and capillaries in the basic case and the control case at *T* = 200.

In the present study, flow-dependent oxygen transport was used instead of the simple treatment of oxygen in our previous studies [24] and many others in which the vessel was treated as point source of oxygen. A test case was designed in which the vessel segment was set as a point source of oxygen. The statistical results in Figure 4 show the proportion of oxygen supply to the tumour tissue by every arterioles order at the end of simulation (*T* = 200). Since the point source of oxygen is only related to the quantity of vessel segments in the test case, the smallest capillaries with largest number provide the highest percentage of oxygen (up to 35%). In the basic case, although the number of larger vessels (arterioles order 1) is the smallest in the four different sized vessels, the abundant blood perfusion allows them to be the primary provider of oxygen, but at limited perfusion locations. There are no noticeable differences between the two cases in the final results of tumour growth. However, flow-dependent oxygen transport can offer more realistic oxygen concentration field since the blood perfusion is known to be heterogeneous in the tumour tissue.

## 4. Discussion and Conclusion

In this work, we have proposed a dynamic mathematical modelling system to investigate the oxygen transport in a three-dimensional preexisting vessel network during the early tumour growth process. A 3D tree-like architecture network with different arterioles orders for vessel diameter is generated as preexisting vasculature in host tissue. To obtain more realistic oxygen concentration field, the advection and diffusion of oxygen in the vessel network are introduced. The computational space is separated into three domains to characterize three distinct physiological processes, which are the oxygen advection equation inside the vessel, the oxygen flux across the vessel wall, and the free oxygen diffusion in the tissue. The oxygen advection inside the vessel and the oxygen flux across the vessel wall are calculated based on the coupling haemodynamic environment including the intravascular blood flow and the interstitial fluid flow. In addition, the dynamic changes of vessel diameter and vessel wall permeability are introduced to reflect a series of pathological characteristics of abnormal blood perfusion in tumours such as vessel dilation, leakage, cooption, remodelling, and collapse.

The simulation focuses on the avascular phase of tumour development and stops before the emergence of angiogenesis phase. The results show the oxygen concentration distribution at different time points of tumour growth. In addition, the influence of preexisting vessel density on the oxygen transport has been discussed. In a case of blood-supply-deficient microenvironment, that is, preexisting vessel network with low MVD, the significant oxygen consumption will lead to the uneven distribution of oxygen concentration through the tumour tissue and eventually upregulate the hypoxia-induced growth factors such as VEGF to activate angiogenesis. Compared with the simple treatment of oxygen transport in which the vessel was treated as point source of oxygen, the modelling of flow-dependent oxygen transport can offer more realistic oxygen concentration field since the blood perfusion is known to be heterogeneous in the tumour tissue. The proposed model not only provides a quantitative approach for investigating the interactions between tumour growth and oxygen delivery, but also is extendable to model other molecules or chemotherapeutic drug transport in the future study.

## Figures and Tables

**Table 1:** Vessel diameter and proportion of three orders of arterioles and capillaries in the model.

	Vessel diameter (*μ*m)	Proportion
Arterioles		
Order 1	50	10%
Order 2	30	20%
Order 3	10	70%
Capillaries	8	NaN

**Table 2:** Parameters of different phenotypes of glioma cells.

Phenotypes	MDE production	Oxygen consumption
Proliferating cells (P)	*μ*_*T*_	*γ*
Quiescent cells (Q)	*μ*_*T*_/5	*γ*/2
Necrotic cells (N)	*μ*_*T*_/10	*γ*/4

**Table 3:** Parameter values used in the simulation.

Parameter	Value	Description	Reference
Δl	10 *μ*m	Lattice constant	
σ_T_	0.82	Average osmotic reflection coefficient for plasma proteins	Baxter and Jain (1989) [19]
π_*v*_	20 mmHg	Colloid osmotic pressure of plasma	Baxter and Jain (1989) [19]
π_i_	15 mmHg	Colloid osmotic pressure of interstitial fluid	Baxter and Jain (1989) [19]
*K*	4.13 × 10^−8^ cm^2^/mmHg s	Hydraulic conductivity coefficient of the interstitium	Baxter and Jain (1989) [19]
*S*/*V*	200 cm^−1^	Surface area per unit volume for transport in the interstitium	Baxter and Jain (1989) [19]
*D*_*m*_	10^−9^ cm^2^s^−1^	MDE diffusion coefficient	Anderson (2005) [14]
*δ*	1.3 × 10^2^ cm^3^M^−1^s^−1^	ECM degradation coefficient	Cai et al. (2011) [17]
*μ*_T_	1.7 × 10^−18^ Mcells^−1^s^−1^	MDE production by TC	Cai et al. (2011) [17]
*μ*_E_	0.3 × 10^−18^ Mcells^−1^s^−1^	MDE production by EC	Cai et al. (2011) [17]
*λ*	1.7 × 10^−8^ s^−1^	MDE decay coefficient	Anderson (2005) [14]
α	1.27 × 10^−15^ *μ*mol/(*μ*m^3^mmHg)	Bunsen solubility coefficient	Fang et al. (2008) [20]
*D*_o_	10^−5^ cm^2^s^−1^	Oxygen diffusion coefficient	Anderson (2005) [14]
*γ*	6.25 × 10^−17^ Mcells^−1^s^−1^	Oxygen consumption coefficient	Anderson (2005) [14]
L_p_^T^	2.8 × 10^−7^ cm/mmHg s	Vessel permeability in tumour tissue	Baxter and Jain (1989) [19]
*E*	6.5 mmHg	Vessel compliance coefficient	Netti et al. (1996) [21]
*b*	0.1	Vessel compliance index	Netti et al. (1996) [21]

## References

[1] Harris A. L. (2002). Hypoxia: a key regulatory factor in tumour growth. *Nature Reviews Cancer*.

[2] Krogh A. (1922). *The Anantomy and Physiology of Capillaries*.

[3] Fukumura D., Jain R. K. (2007). Tumor microvasculature and microenvironment: targets for anti-angiogenesis and normalization. *Microvascular Research*.

[4] Wen P. Y., Kesari S. (2008). Malignant gliomas in adults. *The New England Journal of Medicine*.

[5] Holash J., Maisonpierre P. C., Compton D. (1999). Vessel cooption, regression, and growth in tumors mediated by angiopoietins and VEGF. *Science*.

[6] Vajkoczy P., Farhadi M., Gaumann A. (2002). Microtumor growth initiates angiogenic sprouting with simultaneous expression of VEGF, VEGF receptor-2, and angiopoietin-2. *The Journal of Clinical Investigation*.

[7] Lorger M., Felding-Habermann B. (2010). Capturing changes in the brain microenvironment during initial steps of breast cancer brain metastasis. *The American Journal of Pathology*.

[8] Zhao C., Yang H., Shi H. (2011). Distinct contributions of angiogenesis and vascular co-option during the initiation of primary microtumors and micrometastases. *Carcinogenesis*.

[9] Döme B., Hendrix M. J. C., Paku S., Tóvári J., Tímár J. (2007). Alternative vascularization mechanisms in cancer: pathology and therapeutic implications. *The American Journal of Pathology*.

[10] Goldman D., Popel A. S. (2000). A computational study of the effect of capillary network anastomoses and tortuosity on oxygen transport. *Journal of Theoretical Biology*.

[11] Goldman D., Popel A. S. (2001). A computational study of the effect of vasomotion on oxygen transport from capillary networks. *Journal of Theoretical Biology*.

[12] Tsoukias N. M., Goldman D., Vadapalli A., Pittman R. N., Popel A. S. (2007). A computational model of oxygen delivery by hemoglobin-based oxygen carriers in three-dimensional microvascular networks. *Journal of Theoretical Biology*.

[13] Secomb T. W., Hsu R., Park E. Y. H., Dewhirst M. W. (2004). Green's function methods for analysis of oxygen delivery to tissue by microvascular networks. *Annals of Biomedical Engineering*.

[14] Anderson A. R. A. (2005). A hybrid mathematical model of solid tumour invasion: the importance of cell adhesion. *Mathematical Medicine and Biology*.

[15] Patel A. A., Gawlinski E. T., Lemieux S. ., Gatenby R. A. (2001). A cellular automaton model of early tumor growth and invasion: the effects of native tissue vascularity and increased anaerobic tumor metabolism. *Journal of Theoretical Biology*.

[16] Ferreira S. C., Martins M. L., Vilela M. J. (2002). Reaction-diffusion model for the growth of avascular tumor. *Physical Review E: Statistical, Nonlinear, and Soft Matter Physics*.

[17] Cai Y., Xu S., Wu J., Long Q. (2011). Coupled modelling of tumour angiogenesis, tumour growth and blood perfusion. *Journal of Theoretical Biology*.

[18] Wu J., Xu S., Long Q. (2008). Coupled modeling of blood perfusion in intravascular, interstitial spaces in tumor microvasculature. *Journal of Biomechanics*.

[19] Baxter L. T., Jain R. K. (1989). Transport of fluid and macromolecules in tumors. I. Role of interstitial pressure and convection. *Microvascular Research*.

[20] Fang Q., Sakadžić S., Ruvinskaya L., Devor A., Dale A. M., Boas D. A. (2008). Oxygen advection and diffusion in a three-dimensional vascular anatomical network. *Optics Express*.

[21] Netti P. A., Roberge S., Boucher Y., Baxter L. T., Jain R. K. (1996). Effect of transvascular fluid exchange on pressure-flow relationship in tumors: a proposed mechanism for tumor blood flow heterogeneity. *Microvascular Research*.

[22] Wu J., Long Q., Xu S., Padhani A. R. (2009). Study of tumor blood perfusion and its variation due to vascular normalization by anti-angiogenic therapy based on 3D angiogenic microvasculature. *Journal of Biomechanics*.

[23] Pries A. R., Secomb T. W. (2005). Microvascular blood viscosity in vivo and the endothelial surface layer. *American Journal of Physiology—Heart and Circulatory Physiology*.

[24] Cai Y., Wu J., Xu S., Li Z. (2013). A hybrid cellular automata model of multicellular tumour spheroid growth in hypoxic microenvironment. *Journal of Applied Mathematics*.

